# Parental Media Mediation, Language Ability, and Early Math Ability in Chinese Preschoolers: A Cross-Sectional Study

**DOI:** 10.3390/bs16101913

**Published:** 2026-10-15

**Authors:** Yawei Yang, Minghui Lu, Wenyan Zeng, Jiaqi Wang, Liman Cai

**Affiliations:** 1 School of Education, South China Normal University, Guangzhou 510631, China; 2 School of Digital Media, Shenzhen Polytechnic University, Shenzhen 518055, China

**Keywords:** parental media mediation, language ability, math ability, cross-sectional indirect association, gender differences

## Abstract

Parental media mediation is associated with young children’s cognitive development, but its role in relation to preschoolers’ math ability remains unclear. This study examined whether and how parental media mediation is associated with preschoolers’ math ability, focusing on the cross-sectional indirect association through language ability and whether selected associations differed across study sites and by gender. Participants were 836 preschoolers (*M* age = 4.36, *SD* = 0.87) and their parents from seven kindergartens across three purposively selected study sites in Guangdong, China. Parental media mediation was assessed via parent-report questionnaires; children’s language and math abilities were assessed via one-on-one tests. Results showed that parental media mediation was significantly associated with preschoolers’ math abilities, with a small indirect association from parental media mediation to math ability through overall language ability. The three study sites did not moderate the path from parental media mediation to language ability. Gender did not moderate the mediated associations: although the path from receptive vocabulary to math was stronger for boys than girls, the difference in indirect effects was not significant. These findings provide preliminary evidence that language ability may serve as a link between parental media mediation and preschoolers’ math ability.

## 1. Introduction

Early mathematical competence is one of the strongest predictors of children’s later academic achievement (Duncan et al., 2007/2008). Identifying the family-level factors and cognitive factors associated with preschoolers’ math development is therefore an important task for developmental researchers and educators. As digital media becomes increasingly embedded in young children’ s daily routines, the family media environment has emerged as a significant context for early learning. Within this context, parental media mediation—the strategies parents use to select, monitor, and discuss media content with their children (Clark, 2011)—has been shown to be associated with various aspects of children’s cognitive and language development (Choy et al., 2024; Dore et al., 2018). Yet most existing research has examined the associations between parental media mediation and language or literacy outcomes, while its potential role in relation to mathematical development remains largely unexplored. Moreover, the few studies that have linked media-related parenting to academic outcomes have typically treated the relationship as direct, without investigating the correlates that may help characterize these associations.

Parental media mediation may be associated with richer verbal input through practices such as co-viewing and dialogic interaction around media content, which are associated with vocabulary growth and narrative comprehension. In turn, these improved language abilities may be relevant to mathematics learning, as children rely on linguistic representations to understand number concepts, interpret word problems, and follow procedural instructions (LeFevre et al., 2010; Purpura & Ganley, 2014; Purpura et al., 2011). This suggests an indirect association in which parental media mediation is associated with language development, which subsequently relates to math ability (Hornburg et al., 2024; Yang et al., 2021). Furthermore, this indirect association may vary across children and contexts. The strength of these associations may also vary across children and contexts. The present study therefore examined: (a) the indirect association between parental media mediation and preschoolers’ math ability through language ability and (b) whether the associations differed across study sites and by gender. By doing so, this study aims to advance understanding of how family media practices relate to early mathematical development and to offer empirical reference for differentiated family media education guidance.

### 1.1. Theoretical Framework

This study adopted the DREAMER theory (Dynamic, Relational, Ecological Approach to Media Effects Research; Barr et al., 2024) as its theoretical framework, drawing on two of its core perspectives: relationality and heterogeneity. The relationality perspective holds that media effects on children are embedded in the relational context of parent–child interactions. Accordingly, we proposed an indirect-association model wherein parental media mediation—such as selecting media content, engaging in co-viewing, and providing verbal explanations—enriches the symbolic input children receive, creating favorable conditions for language development. This enhanced language ability, which encompasses crucial resources like symbolic decoding and the integration of visual and linguistic information, is in turn associated with mathematics learning (Hornburg et al., 2024; LeFevre et al., 2010; Yang et al., 2021). This study examines receptive vocabulary and character recognition as two potential intervening language variables. While both are components of language ability, they represent developmentally distinct capacities in preschoolers: oral language naturally precedes written language and serves as a foundational cognitive tool, whereas Chinese character recognition relies heavily on orthography–phonology mapping and visual-spatial processing (D. Lin et al., 2016; Tseng et al., 2023). This differentiation allows for a more nuanced understanding of how parental media mediation may relate to oral versus written language domains, and subsequently, to math ability.

The heterogeneity perspective suggests that media-related associations may vary across individual and contextual factors. Accordingly, we examined study sites as an environmental-level moderator and gender as an individual-level moderator. Regarding study sites, access to high-quality media resources and opportunities for enriched parent–child verbal interaction may vary across contexts. In resource-rich settings, abundant educational media and interactive opportunities may strengthen the link from parental mediation to language ability. In resource-scarce settings, two competing possibilities arise: resource constraints may attenuate this association due to limited access to high-quality content, or parental mediation may instead play a compensatory role, exerting a stronger influence on language development precisely because other enrichment resources are unavailable (Rideout & Robb, 2020; Yuen et al., 2018). Regarding gender, girls tend to develop language skills earlier and show stronger verbal processing advantages during the preschool years (Eriksson et al., 2012), which may render the language-mediated association differently operative for boys and girls. Examining these moderators allows us to delineate for whom and under what conditions the indirect association from parental media mediation to math ability through language ability is stronger or weaker.

### 1.2. Parental Media Mediation and Preschoolers’ Language and Math Abilities

Parental media mediation refers to the ways in which parents guide and regulate children’s engagement with screen media. Lukavská et al. (2021) distinguished three related dimensions: active mediation (communication and guidance during children’s media use), restrictive mediation (rules concerning the amount, content, and circumstances of media use), and overprotection (particularly cautious, protection-oriented control over children’s media experiences). These three dimensions may relate to children’s language and math abilities through two complementary associations. The first is an enrichment association, in which parents directly add cognitive and linguistic input to children’s media experiences through verbal interaction. The second is a regulatory association, in which parents support development not by managing which media children encounter and how much time they spend on screens, thereby shaping the quality of children’s media environments.

The enrichment association is primarily associated with active mediation. When parents discuss, explain, or respond to media content during co-viewing, they connect screen-based representations with children’s existing knowledge and provide linguistic input that children would not receive from media alone. Empirical evidence supports this mechanism for language ability: parental prompts and verbal interaction during television and video viewing facilitate young children’s comprehension and transfer of information from screens (Fender et al., 2010; Strouse et al., 2018); parental involvement during e-book use has been associated with children’s story comprehension (Dore et al., 2018); interactive joint media engagement has been associated with larger vocabulary among younger children (Alroqi et al., 2023); and parental co-engagement during children’s use of literacy applications has been associated with letter knowledge and early writing skills (Neumann, 2018). Evidence for a direct association with math ability comes primarily from structured digital learning contexts. Parent–child interaction during digital math activities can elicit mathematical talk and draw children’s attention to numerical concepts (Zippert et al., 2019). Adult support can facilitate children’s learning within math-focused applications (Berkowitz et al., 2015; Outhwaite et al., 2019). Although these findings support the potential value of active parental involvement for both language and math development, they concern specific forms of parent–child interaction rather than the broader active mediation dimension assessed here.

Theoretically, restrictive mediation and overprotection may represent regulatory aspects of parental media mediation. Rules limiting screen time may preserve opportunities for offline activities involving richer interpersonal interaction, such as shared reading, conversation, and play, while content restrictions may reduce children’s exposure to age-inappropriate material and increase the relative proportion of suitable content (Livingstone & Helsper, 2008; Nikken & Schols, 2015). Similarly, the close supervision characteristic of overprotection may help create a safer and more predictable media environment for preschoolers, who have limited capacity to evaluate media content and regulate their own media use independently (Barr et al., 2024). However, direct evidence linking these specific strategies to language or math performance remains limited.

### 1.3. The Indirect Association Between Parental Media Mediation and Math Ability Through Language Ability

The evidence reviewed above suggests that parental media mediation may be positively associated with both preschoolers’ language ability and math ability. These two associations, however, may not be independent. According to DREAMER theory (Barr et al., 2024), the symbolic input that parents provide during media interactions—including verbal explanations, guided discussion, and exposure to age-appropriate textual content—constitutes an important resource for preschoolers’ language development. Language ability, in turn, is well established as a foundational skill for early mathematical development. Language provides the symbolic decoding and verbal reasoning tools that underpin mathematical thinking (Hornburg et al., 2024; LeFevre et al., 2010). Purpura and Ganley (2014) found that vocabulary predicted multiple early mathematical skills, including number comparison, number sequencing, number identification, and story problem solving, and that preschoolers’ mastery of relational terms such as “more than” and “less than” was directly associated with their capacity to engage in more advanced mathematical activities. More broadly, language ability enables young children to comprehend the linguistic expressions embedded in mathematical problems and to grasp the semantic meaning of mathematical concepts (Purpura et al., 2017). Together, these findings point to a potential indirect association: parental media mediation may relate to math ability not only directly but also indirectly through its role in supporting language development.

However, no study has directly examined whether language ability provides an indirect pathway between parental media mediation and math ability. Existing research investigating parental factors’ association with preschoolers’ math ability has tended to treat language ability as a control variable or a parallel outcome, rather than examining its potential role as an intervening variable (Manolitsis et al., 2013; Skwarchuk et al., 2014). This approach has overlooked the possibility that parenting behaviors relate to math ability through their support of language development. The present study incorporated parental media mediation, language ability, and math ability into a unified analytic framework to address this gap.

### 1.4. Moderation by Study Site and Children’s Gender

The indirect association from parental media mediation to math ability through language ability may not be uniform across all children and contexts. Regarding study site, research in China has documented regional differences in preschool quality, including classroom language and literacy quality, and home learning environments (Rao et al., 2025), preschool education resource allocation (Wang et al., 2024), and digital access and use (Qiu et al., 2023). Such contextual variation raises the possibility that associations involving parental media mediation may differ across locations. However, because study site is an omnibus contextual indicator—each site representing a different city whose unique profile reflects an unknown combination of economic, educational, linguistic, demographic, and technological characteristics—the present study treated between-site differences as an exploratory research question rather than advancing a specific directional hypothesis.

Regarding children’s gender, girls tend to outperform boys in early language abilities such as vocabulary and reading (Below et al., 2010; Reilly et al., 2019) and are more likely to rely on verbal and procedural strategies when solving math problems, whereas boys tend to favor visuospatial approaches (Casey & Ganley, 2021; Gallagher et al., 2000). These differences suggest that language ability may relate to math ability differently for boys and girls. Girls’ greater reliance on verbal strategies may strengthen the language–math link, whereas for boys, whose early language skills tend to be relatively weaker (Robinson & Lubienski, 2011), gains in language ability might yield larger marginal benefits for math performance (Anders et al., 2012). Although gender differences in preschool math achievement appear to be narrowing (Casey & Ganley, 2021), no study has directly examined whether gender moderates the indirect pathway from parental media mediation to math ability via language ability.

### 1.5. The Present Study

Previous research has accumulated substantial empirical evidence on the association between parental media mediation and young children’s cognitive development. However, several gaps remain. First, the potential role of language ability in the association between parental media mediation and preschoolers’ math ability remains insufficiently understood. Most studies have treated language ability and math ability as parallel outcomes (Manolitsis et al., 2013; Yang et al., 2021), without considering the possibility that language ability may serve as an intermediary linking parental media mediation to math ability. Second, although contextual and gender factors have long been recognized in child-development research (Livingstone et al., 2015; Nikken & Jansz, 2013), they are typically treated as control variables or simple predictors rather than as moderators of theoretically specified pathways. Consequently, little is known about whether the association between parental media mediation and children’s language ability varies across study locations or whether the language–math link differs by gender. Third, the existing literature has been predominantly situated in Western contexts, and the extent to which these findings generalize to Chinese families remains unclear (Luo et al., 2025). China’s pronounced regional disparities in digital infrastructure, educational resources, and parenting beliefs (Qiu et al., 2023; Tang & Lan, 2025) make it a particularly informative setting for examining such contextual variation.

To address these gaps, the present study employed a cross-sectional indirect-association model with exploratory moderation analyses to test the associations among parental media mediation, language ability, and preschoolers’ math ability, and to examine whether selected associations differed across study sites and by children’s gender. Situating these analyses in the Chinese context further contributes cross-cultural evidence to a predominantly Western literature and provides empirical grounding for culturally responsive family education guidance. Based on the foregoing review, we proposed the following hypotheses:

**Hypothesis** **1.** *Parental media mediation may be positively associated with preschoolers’ language ability.*

**Hypothesis** **2.** *Parental media mediation may be positively associated with preschoolers’ math ability.*

**Hypothesis** **3.** *Parental media mediation may be indirectly associated with preschoolers’ math ability through language ability in the cross-sectional model.*

**Hypothesis** **4.** *The associations between parental media mediation and language ability may differ across the three study sites. The direction of this moderation is treated as an exploratory question.*

**Hypothesis** **5.** *Children’s gender may moderate the association between language ability and math ability and this moderation is treated as an exploratory question.*

## 2. Materials and Methods

### 2.1. Participants and Procedure

This study employed a multistage recruitment procedure. In the first stage, three study sites were purposively selected to capture variation in regional economic development, operationalized as per capita GDP based on data from the 2023 Economic Bulletin (Guangdong Province Bureau of Statistics, 2024): City A represented a relatively higher per capita GDP level, City B a medium level, and City C a relatively lower level. In the second stage, kindergartens within each city were recruited on a voluntary basis, yielding a total of seven participating institutions. In the third stage, cluster sampling was applied at the classroom level: all classes spanning the junior, middle, and senior preschool years within each kindergarten were included, and all children in those classes who met the inclusion criteria (i.e., normal vision and hearing and no identified developmental delays) and whose parents provided written informed consent were enrolled.

A total of 1190 parent–child dyads were initially invited to participate, of whom 925 parents returned completed questionnaires (response rate = 77.7%). Prior to analysis, 89 questionnaires (9.6% of those returned) were excluded according to the following fixed-order criteria: (a) duplicate or ineligible submissions, including repeated responses for the same child or responses concerning a child outside the eligible age range (*n* = 13); (b) implausibly short completion times of less than 10 min, a threshold corresponding to the lower fifth percentile of the pilot completion-time distribution (*n* = 36); (c) invalid response patterns, defined as selection of the same response option across 10 or more consecutive items or failure of embedded consistency-check items (*n* = 5); and (d) questionnaires discontinued before an entire target scale had been completed (*n* = 35). When a questionnaire met more than one criterion, it was counted under the first applicable one. Because only submission metadata were retained for excluded questionnaires, demographic comparisons between excluded and retained participants could not be conducted. The resulting analytic sample comprised 836 preschoolers (452 boys and 384 girls; *M* age = 4.36, *SD* = 0.87) and their parents, distributed across City A (*n* = 257), City B (*n* = 308), and City C (*n* = 271).

The study protocol was approved by the Ethics Review Committee of the corresponding author’s university. Prior to formal data collection, the research team provided standardized training to the head teachers of the participating classes, who then distributed electronic informed consent forms and self-report questionnaires to parents via Wenjuanxing (Changsha Ranxing Information Technology Co., Ltd., Changsha, China), with assurances that all data would remain strictly confidential. To assess children’s language and mathematics abilities, a dedicated testing team of graduate and undergraduate students was assembled and underwent a two-day standardized training program to ensure strict adherence to the assessment protocols. Upon receiving parental consent, members of this team conducted one-on-one, standardized performance tasks with each child. Parent questionnaires and child assessments were administered during the same data-collection period.

### 2.2. Measures

Parental media mediation was assessed with the 20-item Media Parenting Scale (MEPA-20; Lukavská et al., 2021), which captures three dimensions of parental mediation of children’s screen and media use: active mediation (8 items), restrictive mediation (8 items), and overprotective mediation (4 items). Items cover a range of media types (e.g., television, internet, video games) and are rated on a 5-point Likert scale (1 = strongly disagree, 5 = strongly agree). The MEPA-20 was originally developed through facet-theory-based construction and refined from an initial 36-item pool into a parsimonious 20-item short form (Lukavská et al., 2021); its three-factor structure has since been replicated with evidence of good model fit and strong reliability (Aksoy et al., 2023). For the present study, the MEPA-20 was translated into Chinese and culturally adapted through a multistep procedure. Two bilingual developmental psychology students independently translated the original English items into Chinese, after which a third bilingual researcher compared and reconciled the two forward translations. An independent bilingual translator who had not seen the original instrument then conducted a blind back-translation. The research team compared the back-translated version with the original scale item by item and resolved discrepancies through discussion. Finally, the resulting Chinese version was reviewed by a panel of three early-childhood researchers and three kindergarten teachers, who evaluated each item for clarity, cultural appropriateness, and relevance before the main study commenced.

In the present sample, the scale demonstrated high internal consistency (α = 0.956). Suggestive factor analysis of the proposed three-factor structure yielded CFI = 0.879, TLI = 0.862, RMSEA = 0.077, 90% CI [0.072, 0.082], and SRMR = 0.049. Although the CFI and TLI fell slightly below the conventional 0.90 benchmark, the RMSEA remained below 0.08 and the SRMR was well below 0.06, both meeting widely accepted thresholds for adequate fit (Hu & Bentler, 1999). Given that model fit should be evaluated holistically rather than on the basis of any single index (Marsh et al., 2004), and considering the high internal consistency and the well-established theoretical rationale underlying the three-factor structure (Lukavská et al., 2021; Aksoy et al., 2023), the overall pattern of fit indices was judged to provide acceptable support for the proposed structure. Accordingly, in the primary structural models, the three subscale scores were used as indicators of an overall parental media mediation latent factor.

Language ability was operationalized through two dimensions: receptive vocabulary and Chinese character recognition. Chinese character recognition was measured using the Chinese Character Recognition Task adapted from Shu et al. (2008), which consists of 150 Chinese characters and is appropriate for children aged 3 to 12 years. During testing, children read the characters aloud in sequence, and testing was discontinued if 15 consecutive characters were incorrectly identified. Cronbach’s α for this task was 0.982. Receptive vocabulary was assessed using the Peabody Picture Vocabulary Test. The examiner read a word aloud, and participants selected the corresponding picture from four options. Correct responses scored 1, incorrect 0. Cronbach’s α was 0.984.

Math ability was assessed using the Chinese version of the Test of Early Mathematics Ability, revised by Xie (2014). This test measures nonverbal calculation and mathematical problem-solving abilities. The TEMA is appropriate for children aged 3 to 8 years, and the revised version comprises 120 items. Xie (2014) reported sound psychometric properties, with Cronbach’s α ranging from 0.94 to 0.98 and inter-rater reliability of 0.90. Cronbach’s α was 0.95 in this study.

### 2.3. Data Analysis Strategy

Step 1: Descriptive Statistics, Correlations, and Assumption Checks. Descriptive statistics and bivariate correlations were computed. Univariate normality was evaluated via skewness and kurtosis values, multivariate outliers were screened using Mahalanobis distance (*p* < 0.001), and multicollinearity among the three parental media mediation subscales was examined through inter-correlations and variance inflation factors (VIFs). Score distributions of the child assessments were also inspected for potential ceiling or floor effects.

Step 2: Measurement Model and Measurement Invariance. Prior to testing structural paths, a measurement model comprising three latent factors was specified and evaluated: parental media mediation (indicated by active mediation, restrictive mediation, and overprotective mediation), language ability (indicated by receptive vocabulary and Chinese character recognition), and math ability (indicated by mathematical problem-solving and nonverbal calculation). Model fit and standardized factor loadings were examined to suggest that each construct was adequately represented by its indicators. Subsequently, a series of increasingly restrictive measurement invariance models—configural, metric, scalar, and strict—were tested sequentially across both gender and study site groups. Invariance at each level was evaluated primarily using the change in the Comparative Fit Index (ΔCFI), with a decrement of less than 0.01 serving as the criterion for establishing invariance (Cheung & Rensvold, 2002).

Step 3: Cross-Sectional Indirect-Effect Analyses. Structural equation modeling was employed to test the hypothesized associations. First, the direct associations between parental media mediation and language ability (Hypothesis 1) and between parental media mediation and math ability (Hypothesis 2) were examined. Next, the primary indirect-effect model was specified with parental media mediation as the predictor, overall language ability as the intervening variable, and math ability as the outcome (Hypothesis 3). To further elucidate the specific roles of different language components, the global language intervening variable was subsequently decomposed into two observed indicators—receptive vocabulary and character recognition—and two separate mediation models were estimated: (a) parental media mediation → receptive vocabulary → math ability and (b) parental media mediation → character recognition → math ability. Across all models, children’s age, gender, and study site were included as covariates. Indirect effects were evaluated using bias-corrected bootstrap confidence intervals based on 5000 resamples and were considered statistically significant when the 95% confidence interval excluded zero. Overall model fit was assessed using the chi-square statistic (χ^2^), CFI, Tucker–Lewis Index (TLI), Root Mean Square Error of Approximation (RMSEA), and Standardized Root Mean Square Residual (SRMR), with acceptable fit defined as CFI and TLI ≥ 0.90 and RMSEA and SRMR ≤ 0.08 (Hu & Bentler, 1999).

Step 4: Moderation by study site and children’s gender. The final step examined whether selected constituent paths within the mediation models varied as a function of study site or child gender. Specifically, we tested (a) whether the first-stage path from parental media mediation to language ability differed across the three study sites (Hypothesis 4) and (b) whether child gender moderated the second-stage path from language ability to math ability (Hypothesis 5). Because both moderators were categorical, moderation was tested through multiple-group comparison. For each test, a constrained baseline model in which the target path was fixed to equality across groups was compared against a freed model in which the path was permitted to vary. Nested model comparisons were evaluated with the Satorra–Bentler scaled χ^2^ difference test (Δχ^2^). All models were estimated with the MLR estimator, and standard fit indices (CFI, RMSEA, SRMR) were reported. When study site served as a covariate rather than the grouping variable, it was dummy-coded with City A as the reference group (i.e., City B vs. City A and City C vs. City A). Children’s age was included as a covariate in all models, and continuous predictors were grand-mean centered.

This multiple-group comparison procedure was applied to each of three mediation pathways—overall language ability, receptive vocabulary, and character recognition—crossed with the two moderators (study site and gender), yielding twelve models organized into six paired comparisons. The specific models are described below. Overall language ability as the intervening variable. For the pathway from parental media mediation through overall language ability to math ability, study site differences on the first-stage path (parental media mediation → overall language ability) were tested by comparing a constrained model (M01a) against a freed model (M1a). Gender moderation of the second-stage path (overall language ability → math ability) was then tested by comparing a constrained model (M02a) against a freed model (M2a). Receptive vocabulary as the intervening variable. The same logic was applied to the pathway mediated by receptive vocabulary. Study site differences on the first-stage path (parental media mediation → receptive vocabulary) were tested by comparing a constrained model (M01b) against a freed model (M1b), and gender moderation of the second-stage path (receptive vocabulary → math ability) was tested by comparing a constrained model (M02b) against a freed model (M2b). Character recognition as the intervening variable. Likewise, for the pathway mediated by character recognition, study site differences on the first-stage path (parental media mediation → character recognition) were tested by comparing a constrained model (M01c) against a freed model (M1c), and gender moderation of the second-stage path (character recognition → math ability) was tested by comparing a constrained model (M02c) against a freed model (M2c). In sum, six paired comparisons were conducted across the three intervening variables: three pairs testing whether the first-stage path differed across study sites (M01a vs. M1a, M01b vs. M1b, M01c vs. M1c) and three pairs testing whether gender moderated the second-stage path (M02a vs. M2a, M02b vs. M2b, M02c vs. M2c). For each pair, a significant Satorra–Bentler Δχ^2^ led to retention of the freed model, indicating that the hypothesized moderation was supported; a non-significant result led to retention of the constrained model, indicating no evidence of moderation.

All analyses were performed using SPSS 23.0 (IBM Corp., Armonk, NY, USA) (for descriptive and correlational analyses in Step 1) and Mplus 8.0 (Muthén & Muthén, Los Angeles, CA, USA) (for all latent-variable models in Steps 2–4). Among the 836 retained cases, all variables included in the structural models were completely observed (0% missing); no missing-data imputation or case deletion was therefore required. The choice of estimator varied by analytic purpose: the mediation models in Step 3 were estimated with the maximum likelihood estimator, whereas the measurement invariance models in Step 2 and the moderated mediation models in Step 4 were estimated with the robust maximum likelihood estimator to accommodate potential non-normality and to permit Satorra–Bentler scaled *χ*^2^ difference tests. Notably, children in the present study were recruited from seven kindergartens nested within the three purposively selected study sites, and the data therefore possess a clustered structure. The unconditional intraclass correlation coefficients (ICCs) for the outcome variables ranged from 0.18 to 0.19. These values, however, represent the total between-kindergarten variance prior to the inclusion of any covariates. Because kindergarten membership is largely confounded with study site—a variable that is already included as a fixed covariate in all models—the residual between-kindergarten dependence is likely to be considerably smaller than the unconditional ICCs suggest. With only seven clusters, neither multilevel modeling nor cluster-robust standard error corrections can be expected to perform reliably (Maas & Hox, 2005; Cameron & Miller, 2015), and exploratory attempts to implement such adjustments did not yield stable estimates. Standard errors in the reported models were therefore not adjusted for residual clustering.

## 3. Results

### 3.1. Preliminary Analyses

Table 1 presents the means, standard deviations, skewness, kurtosis, and bivariate correlations among all observed indicators. All indicators met the recommended thresholds for approximate normality (skewness < 3 and kurtosis < 8; Kline, 2016). Child assessment distributions showed no severe ceiling or floor effects. Multivariate outliers were screened via Mahalanobis distance (*p* < 0.001); no influential cases were identified. The three parental media mediation subscales were strongly intercorrelated (*rs* = 0.83–0.86). When entered simultaneously as predictors, their variance inflation factors ranged from 5.94 to 6.90, indicating substantial multicollinearity and producing unstable estimates of their unique associations. Therefore, the primary structural models used the three subscale scores as indicators of an overall parental media mediation latent factor rather than as simultaneous strategy-specific predictors. This modeling decision addresses the empirical overlap among the subscales but does not imply that active mediation, restrictive mediation, and overprotection are conceptually equivalent.

Correlation analyses further revealed that indicators of parental media mediation were positively correlated with indicators of preschoolers’ language ability (*rs* = 0.070–0.102, *p* < 0.05) and math ability (*rs* = 0.108–0.139, *p* < 0.01). By contrast, preschoolers’ language ability indicators were positively correlated with math ability indicators (*rs* = 0.512–0.626, *p* < 0.01). Children’s age showed strong positive correlations with both language ability indicators (*rs* = 0.521–0.666) and math ability indicators (*rs* = 0.584–0.617). Gender was related to children’s receptive vocabulary (*rs* = 0.079, *p* < 0.05).

### 3.2. Pathway Measurement Model and Invariance

#### 3.2.1. Measurement Model

Before testing the structural model, we evaluated the measurement model comprising three latent factors—parental media mediation (indicated by active, restrictive, and overprotection subscales), language ability (receptive vocabulary and Chinese character recognition), and math ability (mathematical problem-solving and nonverbal calculation). The model fit the data well, χ^2^ = 6.269, *df* = 11, CFI = 1.00, TLI = 1.002, RMSEA = 0.000, SRMR = 0.008. All standardized factor loadings were significant (*p* < 0.001) and ranged from 0.657 to 0.956, indicating that each latent construct was adequately measured by its indicators. Complete factor loadings and residual variances are reported in Table 2.

#### 3.2.2. Measurement Invariance Across Gender and Study Sites

Configural, metric, scalar, and strict measurement invariance were tested sequentially across gender and study site groups prior to cross-group structural comparisons. Across gender, the metric invariance model showed excellent fit (*χ*^2^ = 21.423, *df* = 26, CFI = 1.000, TLI = 1.002, RMSEA = 0.000, SRMR = 0.012), with a ΔCFI of 0.000 relative to the configural model, well below the 0.01 threshold. The scalar invariance model showed good fit (*χ*^2^ = 32.812, *df* = 30, CFI = 0.999, TLI = 0.999, RMSEA = 0.015, *SRMR* = 0.017), with a ΔCFI of 0.001 relative to the metric model, supporting scalar invariance. The strict invariance model showed acceptable fit (*χ*^2^ = 54.572, *df* = 37, CFI = 0.995, TLI = 0.995, RMSEA = 0.034, SRMR = 0.036), with a ΔCFI of 0.004, supporting strict invariance. Across study sites, the metric invariance model showed excellent fit (*χ*^2^ = 30.416, *df* = 41, CFI = 1.000, TLI = 1.004, RMSEA = 0.000, SRMR = 0.021), with a ΔCFI of 0.000. The scalar invariance model showed acceptable fit (*χ*^2^ = 64.024, *df* = 49, CFI = 0.996, TLI = 0.995, RMSEA = 0.033, SRMR = 0.029), with a ΔCFI of 0.004, supporting scalar invariance. The strict invariance model showed acceptable fit (*χ*^2^ = 80.288, *df* = 63, CFI = 0.995, TLI = 0.995, RMSEA = 0.031, SRMR = 0.036), with a ΔCFI of 0.001, supporting strict invariance. These results suggest that configural, metric, scalar, and strict measurement invariance were established across gender and study site groups, providing a robust basis for unbiased cross-group structural comparisons and subsequent multiple-group comparison sites (Vandenberg & Lance, 2000; Putnick & Bornstein, 2016).

### 3.3. Indirect-Association Analyses

Before estimating the mediation model, we tested Hypotheses 1 and 2 using two separate structural equation models. The first model examined the association between parental media mediation and preschoolers’ language ability while controlling for age, gender, and study site. This model showed excellent fit *(χ*^2^ = 27.999, *df* = 16, CFI = 0.996, TLI = 0.993, SRMR = 0.018, RMSEA = 0.030), and parental media mediation was non-significantly associated with language ability (*β* = 0.052, 95% CI [0.000, 0.104], *p* = 0.052); therefore, Hypothesis 1 was not supported. The second model examined the association between parental media mediation and preschoolers’ math ability while controlling for the same covariates. This model also showed excellent fit (*χ*^2^ = 23.071, *df* = 16, CFI = 0.998, TLI = 0.996, SRMR = 0.011, RMSEA = 0.023), and parental media mediation was positively associated with math ability before overall language ability was included as an intervening variable (*β* = 0.072, *p* < 0.05), supporting Hypothesis 2.

Subsequently, the mediation model demonstrated good fit (*χ*^2^ = 95.317, *df* = 31, CFI = 0.988, TLI = 0.980, RMSEA = 0.050, SRMR = 0.034) The bias-corrected bootstrapped 95% confidence interval (CI) for the indirect effect (*β* = 0.048) did not contain zero [0.002, 0.100], indicating a significant indirect association. The direct association from parental media mediation to math ability was no longer significant (*β* = 0.020, 95% CI [−0.040, 0.068]). To explore the specific contributions of language dimensions, separate models were specified for receptive vocabulary (PPVT) and character recognition (CR), and the results are summarized in Table 3. The PPVT model demonstrated good fit (*χ*^2^ = 34.393, *df* = 23, CFI = 0.998, TLI = 0.996, RMSEA = 0.024, SRMR = 0.029). The indirect association through receptive vocabulary was not statistically significant (*β* = 0.019, 95% CI [0.000, 0.040]), because the lower confidence limit did not exclude zero. The direct association became non-significant (*β* = 0.050, 95% CI [−0.006, 0.102]). Conversely, the CR model also showed good fit (*χ*^2^ = 36.819, *df* = 23, CFI = 0.997, TLI = 0.995, RMSEA = 0.027, SRMR = 0.028), but the indirect effect (*β* = 0.013) was not significant as the 95% CI contained zero [−0.006, 0.032]. The direct association remained significant (*β* = 0.056, 95% CI [0.001, 0.111]). These findings indicate that a small cross-sectional indirect association was statistically detectable only when language ability was modeled as an overarching latent factor. Neither receptive vocabulary nor character recognition yielded a statistically significant specific indirect effect. Thus, Hypothesis 3 received only provisional support at the latent-factor level, and the observed association cannot be attributed to a particular language dimension.

### 3.4. Moderation by Study Site and Children’s Gender

Table 4 presents the model fit statistics and Δ*χ*^2^*_SB_* difference tests for the six paired model comparisons across the three intervening variables. Results are organized by hypothesis below.

When overall language ability served as the intervening variable, the constrained model (M01a; *χ*^2^ = 104.324, *df* = 85, RMSEA = 0.029, CFI = 0.996, SRMR = 0.044) fit the data well, and freeing the first-stage path across the three study sites (M1a) did not significantly improve fit (Δ*χ*^2^*_SB_* = 1.16, *p* = 0.561). The same pattern held when receptive vocabulary served as the intervening variable (M01b vs. M1b: Δ*χ*^2^*_SB_* = 1.93, *p* = 0.381) and when character recognition served as the intervening variable (M01c vs. M1c: Δ*χ*^2^ = 0.14, *p* = 0.933). Across all three intervening variables, the association between parental media mediation and children’s language outcomes did not differ significantly across study sites. Hypothesis 4 was therefore not supported.

When overall language ability served as the intervening variable, the constrained model (M01a; *χ*^2^ = 104.324, *df* = 85, RMSEA = 0.029, CFI = 0.996, SRMR = 0.044) fit the data well, and freeing the first-stage path across the three study sites (M1a) did not significantly improve fit (Δ*χ*^2^*_SB_* = 1.16, *p* = 0.561). The same pattern held when receptive vocabulary served as the intervening variable (M01b vs. M1b: Δ*χ*^2^*_SB_* = 1.93, *p* = 0.381) and when character recognition served as the intervening variable (M01c vs. M1c: Δ*χ*^2^ = 0.14, *p* = 0.933). Across all three intervening variables, the association between parental media mediation and children’s language outcomes did not differ significantly across study sites. Hypothesis 4 was therefore not supported.

When overall language ability served as the intervening variable, the constrained model (M02a; *χ*^2^ = 124.869, *df* = 63, RMSEA = 0.048, CFI = 0.987, SRMR = 0.043) fit well, and freeing the second-stage path across boys and girls (M2a) did not significantly improve fit (Δ*χ*^2^*_SB_* = 0.30, *p* = 0.586). When character recognition served as the intervening variable, the result was the same (M02c vs. M2c: Δ*χ*^2^*_SB_* = 0.05, *p* = 0.821). A different pattern emerged for receptive vocabulary. Freeing the receptive vocabulary → math path across gender groups (M2b) significantly improved model fit over the constrained model (M02b; Δ*χ*^2^*_SB_* = 7.12, *p* = 0.008), indicating that child gender moderated this association: the path was stronger for boys (b = 0.42) than for girls (b = 0.26). However, this path-level gender difference did not propagate through the full mediation chain. The conditional indirect effect was not statistically significant for either boys (95% CI [−0.001, 0.045]) or girls (95% CI [−0.001, 0.028]), and the index of moderated mediation suggested that the two conditional indirect effects did not differ significantly (Δab = 0.008, 95% CI [−0.003, 0.019]). In summary, Hypothesis 5 was partially supported at the path level—gender moderated the receptive vocabulary → math association—but moderated mediation was not supported.

## 4. Discussion

This study examined the cross-sectional associations among parental media mediation, language ability, and preschoolers’ math ability, as well as whether selected associations differed across study sites and by gender. The findings can be summarized as follows. Parental media mediation was significantly associated with preschoolers’ math ability but not with language ability. A small indirect association from parental media mediation to math ability through overall language ability was statistically detectable. When the language construct was decomposed into receptive vocabulary and character recognition, neither yielded a significant specific indirect effect. The associations between parental media mediation and the three language measures did not differ significantly across the three study sites. Regarding gender, the path from receptive vocabulary to math ability was stronger for boys than for girls, but this path-level difference did not result in significantly different indirect effects; gender did not moderate the mediated pathway through any of the three language measures.

### 4.1. Parental Media Mediation and Preschoolers’ Language and Math Ability

Parental media mediation was significantly associated with preschoolers’ math ability, supporting Hypothesis 2. By contrast, the association between parental media mediation and language ability was marginally non-significant, and Hypothesis 1 was not supported. That the association with language ability did not reach statistical significance (*β* = 0.052, *p* = 0.052) deserves consideration. The effect was in the expected positive direction and only marginally non-significant, suggesting that the absence of a statistically detectable association may partly reflect limited statistical power—particularly given that standard errors were not adjusted for clustering—rather than the complete absence of a relationship. It remains plausible that parental media mediation contributes modestly to children’s language development, but the present data do not provide sufficient evidence to support this claim. The significant math association, though small in magnitude, should not be dismissed from a developmental perspective. Media mediation practices—such as discussing screen content, selecting programs, or setting usage limits—are repetitive and cumulative in nature. Over time, these continuous micro-interactions may function as a supportive developmental scaffold, incrementally enriching children’s symbolic environment and priming them for formal learning. Consequently, even small improvements in mediation quality, when sustained across early childhood, can potentially translate into meaningful benefits at the population level. This finding is consistent with prior research demonstrating the subtle but positive role of parental involvement in children’s media consumption (e.g., Alroqi et al., 2023; Dore et al., 2018; Strouse et al., 2018).

Several processes drawn from prior literature may help explain why parental media mediation could plausibly relate to both language and math outcomes, and thus provide a theoretical rationale for the associations tested in this study—even though only the math association reached significance in the present sample. At the content level, parents who actively mediate may enhance children’s learning input by selecting high-quality educational media that exposes children to rich language models supporting vocabulary and comprehension (Fender et al., 2010), as well as early mathematical content such as numerical concepts and counting principles (Berkowitz et al., 2015). At the interaction level, joint media use may help children bridge screen-based symbols and real-world meanings through naming, questioning, and explaining, thereby deepening language comprehension (Roseberry et al., 2014) and rendering abstract numerical concepts more concrete (Gunderson & Levine, 2011; Zippert et al., 2019). At the regulation level, appropriate restrictive mediation may preserve time for alternative learning activities such as shared reading (Livingstone et al., 2015) and promote more focused engagement with educational content (Dore et al., 2018). These processes are drawn from prior empirical work and provide a conceptual basis for the hypothesized associations; they are not offered as suggested explanations of the present results. Whether these mechanisms account for the significant math association—or for a language association that the present study lacked the power to detect—remains an open question. It should be noted, however, that parental media mediation in this study was measured as three broad, self-reported dimensions—active mediation, restrictive mediation, and overprotection—rather than directly capturing real-time parent–child interactions during media use. The specific behaviors discussed above therefore represent hypothesized explanatory processes rather than directly observed mechanisms in the present dataset. Future research using observational methods or daily diary designs is needed to evaluate these micro-level processes.

### 4.2. The Role of Language Ability in the Indirect Association

The primary indirect-association model yielded a small but statistically significant indirect effect from parental media mediation to math ability through overall language ability, providing provisional support for Hypothesis 3. When language ability was decomposed into its two specific components, however, neither the pathway through receptive vocabulary nor the pathway through character recognition reached statistical significance. This pattern—significant at the latent-factor level but not at the specific-dimension level—is not inherently contradictory. The latent language factor captures the shared variance common to both receptive vocabulary and character recognition, thereby reducing measurement error and providing a more reliable and comprehensive estimate of the overall language construct. Each specific dimension alone carries unique measurement error and represents only part of the broader language ability, which limits its statistical power as an individual mediator. Nonetheless, because the first-stage path from parental media mediation to language ability was not itself statistically significant (*β* = 0.052, *p* = 0.052) and the lower bound of the indirect-effect confidence interval was very close to zero, the indirect association should be regarded as fragile. Replication with larger samples, a greater number of clusters, and longitudinal measurement is needed before any mediational interpretation can be sustained.

From a theoretical perspective, DREAMER theory (Barr et al., 2024) provides a framework for understanding why language ability may serve as a link between parental media mediation and math ability. Parental media mediation practices such as content selection, joint engagement, and verbal explanation may enrich children’s symbolic and linguistic input (Nikken & Jansz, 2013; Strouse et al., 2018). This enriched input may in turn support the development of language comprehension—the ability to understand word meanings, relational terms, and sentence structures—which provides the representational tools that children need to interpret the linguistic expressions embedded in mathematical problems and to grasp mathematical concepts (Hornburg et al., 2024; Purpura & Ganley, 2014; Purpura et al., 2017). Whereas existing research has tended to treat language and math abilities as parallel outcomes of family factors (Rao et al., 2025), the present study adopted a sequential framework positioning language ability as a potential intervening variable. This approach extends DREAMER theory’s proposition that parental relational scaffolding within the family media ecology may support children’s cognitive development through enriched symbolic input (Barr et al., 2024), providing initial correlational evidence that language ability may serve not only as a co-occurring outcome of family media practices but also as a pathway through which those practices relate to mathematical development.

The finding that character recognition did not yield a significant specific indirect effect, whereas the pathway through receptive vocabulary approached significance, is also theoretically informative. Character recognition is primarily a code-based decoding skill that, at the preschool stage, relies heavily on orthography–phonology mapping and visual-spatial processing (D. Lin et al., 2016; Tseng et al., 2023) and may not yet serve the same bridging function for mathematical reasoning. Receptive vocabulary, by contrast, is a meaning-based skill that more directly supports the semantic comprehension required for understanding number concepts and mathematical language. This distinction suggests that, to the extent that language ability bridges parental media mediation and math ability, the meaning-based dimension of language may be more relevant than the code-based dimension—although the present data can only suggest this possibility, as neither specific pathway reached statistical significance.

It is important to situate these findings within the broader home learning ecology. Parental media mediation does not occur in isolation; rather, it is likely embedded within a constellation of home learning practices—including home literacy activities, shared book reading, and parent math talk—that have each been shown to predict children’s language and mathematical development (Manolitsis et al., 2013; Skwarchuk et al., 2014; Levine et al., 2010/2011). These dimensions of the home environment tend to co-occur, as parents who invest in one form of cognitive stimulation typically invest in others as well (Niklas & Schneider, 2017; Skwarchuk et al., 2014), possibly reflecting a common underlying orientation toward active engagement with children’s learning that is often closely tied to family socioeconomic factors such as parental education. Parental media mediation may therefore function as one manifestation of this broader orientation: parents who scaffold their children’s media use are likely to be the same parents who read frequently with their children and engage in rich mathematical conversation during daily routines (Nikken & Schols, 2015; Zippert et al., 2019). From this perspective, the indirect association identified in the present study may partially reflect the contribution of this broader home learning ecology, as well as underlying factors like parental education, rather than solely the unique association of media-specific parenting. This interpretation does not negate the theoretical significance of the finding, as the role of language ability in the cross-sectional indirect association remains informative regardless of whether media mediation operates uniquely or in concert with other home learning practices.

### 4.3. Study Site Differences and Children’s Gender

The associations between parental media mediation and the three language measures—overall language ability, receptive vocabulary, and character recognition—did not differ significantly across the three study sites; Hypothesis 4 was therefore not supported. Previous research has documented regional disparities in family and preschool environments across China (Rao et al., 2025; Wang et al., 2024), which provided a rationale for examining cross-site variation. Two considerations may help account for the absence of statistically detectable differences. First, the increasing penetration of digital technology across Chinese regions may have reduced disparities in families’ access to media devices and opportunities for media-related co-engagement. Smartphone and internet access have become nearly universal across urban and semi-urban areas in China, potentially narrowing the digital divide among regions that differ in level of economic development (China Internet Network Information Center [CNNIC], 2025; Gou & Dezuanni, 2018; W. Lin & Feng, 2021). If families across the three study sites now have broadly comparable access to devices and digital content, between-site differences may not translate into meaningful differences in the conditions under which media mediation relates to children’s language outcomes. Second, the null finding may reflect a design limitation rather than a substantive conclusion. The three study sites were selected to represent different levels of economic development, but this between-city distinction does not index the within-city, family-level variation in income, education, or digital resources that is more proximally relevant to children’s home media experiences (Gou & Dezuanni, 2018; Rideout & Robb, 2020). Moreover, because each level of economic development corresponds to a single purposively selected city, the study site is confounded with other city-specific characteristics such as local education resources, dialect environment, and digital infrastructure. This null finding therefore indicates only that no statistically detectable differences were observed among the three study sites and should not be interpreted as definitive evidence that regional economic conditions do not moderate these associations.

Regarding gender, the moderated mediation analyses yielded a nuanced pattern of results. Gender did not moderate the path from overall language ability to math ability, nor did it moderate the path from character recognition to math ability. However, the path from receptive vocabulary to math ability was significantly stronger for boys (b = 0.42) than for girls (b = 0.26), partially supporting Hypothesis 5 at the path level. This finding may appear counterintuitive, given that girls typically demonstrate earlier and more advanced verbal development during the preschool years (Bornstein et al., 2004; Lange et al., 2016). However, precisely because girls tend to develop receptive vocabulary earlier and more uniformly, there may be less inter-individual variability in vocabulary among girls at this age. Reduced variability constrains the magnitude of statistical associations, meaning that vocabulary differences among girls may carry less predictive power for math ability—not because vocabulary is less functionally important for girls, but because there is simply less variation to drive the association. For boys, whose receptive vocabulary tends to be more variable during the preschool years, individual differences in vocabulary may be more consequential for math performance, thereby strengthening the vocabulary–math link (Robinson & Lubienski, 2011). Importantly, however, this path-level gender difference did not propagate through the full indirect-association chain. The conditional indirect effects were not statistically significant for either boys or girls, and the index of moderated mediation suggested that the two conditional indirect effects did not differ significantly. Thus, moderated mediation was not supported: although gender moderated one constituent path, it did not produce meaningfully different indirect pathways from parental media mediation to math ability for boys and girls. The absence of gender moderation on the character recognition → math path may reflect the nature of the skill itself. Chinese character recognition—situated within a logographic writing system—relies on visual–orthographic processing that is distinct from oral vocabulary comprehension and may be less susceptible to gender-related differences in verbal processing strategies (D. Lin et al., 2016; McBride-Chang et al., 2005; Shu et al., 2006).

### 4.4. Contributions, Limitations, and Future Directions

The present study makes three contributions to the literature. First, by incorporating parental media mediation, language ability, and math ability into a unified analytic framework, this study provides preliminary evidence that language ability may function as a pathway linking parental media mediation to preschoolers’ math ability. Although this indirect association was small and provisional, the sequential modeling approach itself represents a conceptual advance that is consistent with DREAMER theory’s relational perspective (Barr et al., 2024). Second, the exploratory multi-site comparison found no statistically detectable differences in the focal associations across three sampled locations in Guangdong. While this does not constitute definitive evidence about socioeconomic moderation, it extends the empirical evidence base to multiple locations within a Chinese context that is underrepresented in the existing literature. Third, the finding that gender moderated the receptive vocabulary → math path is consistent with DREAMER theory’s emphasis on heterogeneity in media-related associations (Barr et al., 2024) and highlights that the language–math link may operate differently depending on the variability structure of children’s language skills.

Regarding practical implications, the most robust and directly actionable finding is the strong association between children’s language ability and early math ability. This pattern suggests that early numeracy support in Chinese preschools may benefit from sustained attention to children’s language competence, particularly their comprehension of the vocabulary and relational terms embedded in mathematical activities. This interpretation is consistent with previous intervention research showing that mathematically relevant language can be incorporated into dialogic learning activities to support early numeracy development (Purpura et al., 2017). Beyond this direct association, the indirect pathway from parental media mediation through language ability to math ability—though modest in magnitude—carries practical relevance. It suggests that parental media mediation may not only support children’s cognitive outcomes directly but may also do so indirectly by fostering the language skills that underpin mathematical reasoning. For practitioners and parent educators, this implies that media mediation guidance need not focus exclusively on limiting screen time or selecting educational content; it may be equally important to encourage verbal interactions around media—such as naming, explaining, and questioning—that enrich children’s language environment. Such language-enriching mediation practices could, over time, contribute to a stronger linguistic foundation that in turn facilitates early mathematical learning. That said, these indirect pathways should be interpreted with appropriate caution. The MEPA-20 did not separately assess specific behaviors such as co-viewing frequency, content selection, verbal scaffolding, or screen-time duration. The present results therefore point to the potential value of media mediation as a developmental resource, but do not yet specify which particular mediation behaviors matter most or under what conditions they are most effective. Future research using behavior-specific measures that can separately assess the frequency, content, duration, and quality of parent–child media interactions is needed to refine these practical recommendations. Importantly, because the present study is cross-sectional, all practical implications reflect correlational patterns rather than established causal effects. Longitudinal and experimental designs are needed before these findings can be translated into specific recommendations for families or early-childhood practitioners.

Several limitations qualify these findings and point to future research directions. First, because all variables were measured concurrently, temporal precedence cannot be established; the indirect effects reported here are statistical decompositions of cross-sectional covariance and do not constitute evidence of mediation. Reverse or reciprocal orderings—for example, stronger early numeracy eliciting more parental engagement with media content—are equally consistent with these data, and longitudinal or experimental designs are required to test the proposed process.

Second, children were recruited from seven kindergartens located in three purposively selected sites, and the data are therefore clustered. The unconditional intraclass correlations were substantial (≈0.18–0.19), although a large share of this between-kindergarten variance is likely absorbed by the site covariate included in all models. Standard errors were nevertheless not adjusted for the residual clustering. With only seven clusters, neither multilevel estimation nor cluster-robust correction can be expected to perform reliably (Maas & Hox, 2005; Cameron & Miller, 2015), and exploratory attempts to fit such models did not yield stable estimates. Replication in designs with a substantially larger number of clusters (e.g., ≥50) and measured school- and family-level covariates is needed before they can be evaluated with confidence.

Third, several measurement limitations should be acknowledged. The structural validity of the Chinese MEPA-20 received mixed support: although the RMSEA and SRMR were acceptable, the CFI (0.879) and TLI (0.862) fell below 0.90. We refrained from adding post hoc residual correlations to avoid capitalizing on sample-specific variation, but a dedicated validation study is needed to examine item redundancy and optimize the measurement structure. Parental media mediation was modeled as a single composite rather than being disaggregated into active mediation, restrictive mediation, and overprotection. Although these strategies are conceptually distinct, the three subscales could not be empirically separated in the present sample due to severe collinearity when entered simultaneously and confounded total effects when modeled individually—attributable in part to shared method variance from a single parent-report item block. Isolating strategy-specific contributions will require observational, multi-informant, or experimental designs. In addition, the study assessed only broad media mediation rather than math-specific media engagement such as co-using math applications or selecting math-oriented programs; future research should examine both general and targeted practices to determine whether math-specific engagement has a stronger predictive role.

Fourth, several potentially important variables were not included in the models due to data collection constraints. At the family level, these include parental education, occupation, household income, educational beliefs, broader parenting style, home literacy and numeracy activities, parent–child interaction quality, parental digital literacy, household media device access, and the quality of media content children were exposed to. At the child level, executive functioning and exact screen time duration and content type were also unavailable. Because these factors may be associated with both parental media mediation and children’s cognitive abilities, their omission introduces the possibility of residual confounding and omitted-variable bias. Relatedly, the three study sites were selected to represent different levels of economic development, but each level was represented by only one city. Each study site therefore captured an unknown mixture of city-level characteristics—including local curriculum and teacher quality, dialect and language environment, digital infrastructure, migration composition, and screen-use norms—and should not be equated with socioeconomic status. Future research aiming to test socioeconomic moderation should include multiple cities within each economic development stratum and, ideally, incorporate direct measures of socioeconomic conditions at both the family level, such as parental education, household income, and the home learning environment, and the community level, such as neighborhood deprivation indices.

## 5. Conclusions

In this multi-site sample of Chinese preschoolers, parental media mediation showed a small, indirect association with math ability that operated through overall language ability. These results suggest that, within the broader home learning environment, how parents guide children’s media use may have a modest, language-mediated link to early numeracy—even after accounting for study site and child gender. The findings should be interpreted with caution, however, given the small effect size, the cross-sectional design, and the absence of controls for family socioeconomic resources and school-level clustering.

## Figures and Tables

**Table 1:** Means, Standard Deviations, and Correlations Among Study Variables.

Variable	*M*	*SD*	Skewness	Kurtosis	Range	1.	2.	3.	4.	5.	6.	7.	8.
1. Active mediation	25.165	5.962	−0.264	0.442	8–40	1							
2. Restrictive mediation	22.402	5.471	−0.184	0.780	7–35	0.857 **	1						
3. Overprotection	12.821	3.384	−0.200	0.415	3–20	0.829 **	0.842 **	1					
4. Receptive vocabulary	79.275	36.274	0.687	0.362	11–200	0.090 **	0.070 *	0.077 *	1				
5. Character recognition	11.429	17.460	2.428	6.200	0–116	0.102 **	0.077 *	0.091 **	0.541 **	1			
6. Nonverbal calculation	4.496	2.373	0.586	−0.248	0–10	0.136 **	0.120 **	0.109 **	0.539 **	0.512 **	1		
7. Problem-solving	4.440	3.909	0.989	0.123	0–16	0.139 **	0.110 **	0.108 **	0.626 **	0.577 **	0.670 **	1	
8. Age	4.358	0.870	0.256	−0.428	2–7	0.080 *	0.079 *	0.063	0.666 **	0.521 **	0.584 **	0.617 **	1
9. Gender	0.459	0.499	0.164	−1.978	−	−0.004	−0.010	−0.010	0.079 *	0.023	−0.047	0.011	0.005

Note. * *p* < 0.05, ** *p* < 0.01 (two-tailed).

**Table 2:** Measurement Model Results.

Latent Variable	Indicator	Std. Loading	*p*-Value	Residual Variance	Variance Explained (R^2^)
Math Ability	problem-solving	0.938	<0.001	0.121	0.879
	nonverbal calculation	0.821	<0.001	0.326	0.674
Language Ability	receptive vocabulary	0.763	<0.001	0.418	0.582
	Chinese character recognition	0.657	<0.001	0.568	0.432
Parental Media Mediation	active mediation	0.939	<0.001	0.119	0.881
	restrictive mediation	0.956	<0.001	0.087	0.913
	overprotection	0.937	<0.001	0.122	0.878

Note. All factor loadings are standardized estimates and significant at *p* < 0.001. Variance explained (*R*^2^) = standardized loading^2^. Residual variance = 1 − *R*^2^.

**Table 3:** Summary of Language Models (receptive vocabulary vs. character recognition).

Model	Indirect Effect (Estimate)	95% CI	Direct Effect (c′)	95% CI
Parental media mediation→receptive vocabulary→math ability	0.019	[0.000, 0.040]	0.050	[−0.006, 0.102]
Parental media mediation→character recognition→math ability	0.013	[−0.006, 0.032]	0.056	[0.001, 0.111]

Note. CIs are bias-corrected bootstrap 95% confidence intervals based on 5000 resamples.

**Table 4:** Model Fit and Satorra–Bentler Scaled *χ*^2^ Difference Tests across Three Indirect-Association Models.

Model	*c* (*χ*^2^/*df*)	*P*(Model *χ*^2^)	*χ* ^2^	*df*	RMSEA	CFI	SRMR	Moderation Test
Overall language as intervening variable								
M01a	1.227	0.076	104.324	85	0.029	0.996	0.044	_
M1a	1.242	0.067	103.108	83	0.029	0.996	0.045	Δ*χ*^2^_SB_ = 1.16, *p* = 0.561
M02a	1.982	<0.001	124.869	63	0.048	0.987	0.043	_
M2a	2.014	<0.001	124.892	62	0.049	0.987	0.042	Δ*χ*^2^_SB_ = 0.30, *p* = 0.586
Receptive vocabulary as intervening variable								
M01b	0.786	0.893	49.498	63	0.000	1.000	0.042	_
M1b	0.780	0.895	47.595	61	0.000	1.000	0.044	Δ*χ*^2^_SB_ = 1.93, *p* = 0.381
M02b	1.528	0.011	71.793	47	0.036	0.994	0.042	
M2b	1.4	0.038	64.401	46	0.031	0.996	0.041	Δ*χ*^2^_SB_ = 7.12, *p* = 0.008
Character recognition as intervening variable								
M01c	0.820	0.845	51.688	63	0.000	1.000	0.041	_
M1c	0.840	0.810	51.215	61	0.000	1.000	0.041	Δ*χ*^2^_SB_ = 0.14, *p* = 0.933
M02c	1.114	0.273	52.374	47	0.017	0.999	0.040	_
M2c	1.15	0.225	52.896	46	0.019	0.998	0.040	Δ*χ*^2^_SB_ = 0.05, *p* = 0.821

## Data Availability

The datasets used and analyzed during the current study are available from the corresponding author on reasonable request. The data are not publicly available due to privacy and ethical restrictions.

## Abbreviations

The following abbreviations are used in this manuscript:

AIC	Akaike information criterion
CFI	Comparative fit index
CI	Confidence interval
DREAMER	Dynamic, Relational, Ecological Approach to Media Effects Research
LRT	Likelihood ratio test
MEPA-20	Media Parenting Scale
RMSEA	Root mean square error of approximation
SEM	Structural equation modeling
SPSS	Statistical Package for the Social Sciences
SRMR	Standardized root mean square residual
TEMA	Test of Early Mathematics Ability
TLI	Tucker–Lewis index
